# A rare cause of chronic dysphagia: pulmonary inflammatory myofibroblastic tumor with distal esophagus invasion

**DOI:** 10.1186/s13019-021-01662-0

**Published:** 2021-10-09

**Authors:** Yi-Min Gu, Long-Qi Chen

**Affiliations:** Department of Thoracic Surgery, West China Hospital of Sichuan University, No. 37, Guoxue Alley, 610041 Chengdu, China

**Keywords:** IMT, Esophagus invasion, Chronic dysphagia, Treatment

## Abstract

**Background:**

Inflammatory myofibroblastic tumor (IMT) is rare intermediate tumor, which happens mostly in children and young adults.

**Case presentation:**

Reported is the successful treatment of a 29-year-old man presented with progressively dysphagia and weight loss. No other abnormal symptoms were observed. The contrast enhanced computed tomography (CT) revealed a dumbbell-shaped lesion between lung and esophagus. Finally, it was pathologically diagnosed as pulmonary IMT invading to the distal esophagus after operation. The patient underwent partial esophagectomy and left lower lobectomy, and was discharged on 10th postoperative day.

**Conclusions:**

IMT is a rare lesion that usually occurs in the lung, but pulmonary IMT with distal esophagus invasion has not been described previously. Discriminating untypical symptom, completed resection, pathological expertise and closed follow-up will reach the successful diagnosis and treatment.

## Introduction

IMT is rare mesenchymal tumor [1–3], which happens mostly in children and young adults [4]. It is regarded as intermediate tumor because of a rare possibility of recurrence [5]. The symptoms or imaging findings of IMT vary from its location and histological evidence can be of great help on diagnosis. Here we presented a 29-year-old male patient with extremely rare pulmonary IMT invading to distal esophagus, with the clearly dumbbell-shaped lesion showed on the contrast enhanced CT scan. Definite diagnosis and satisfied management were achieved by completed resection and routine follow-up. The literatures correlated with IMT were also reviewed.

## Case presentation

**Fig. 1:**
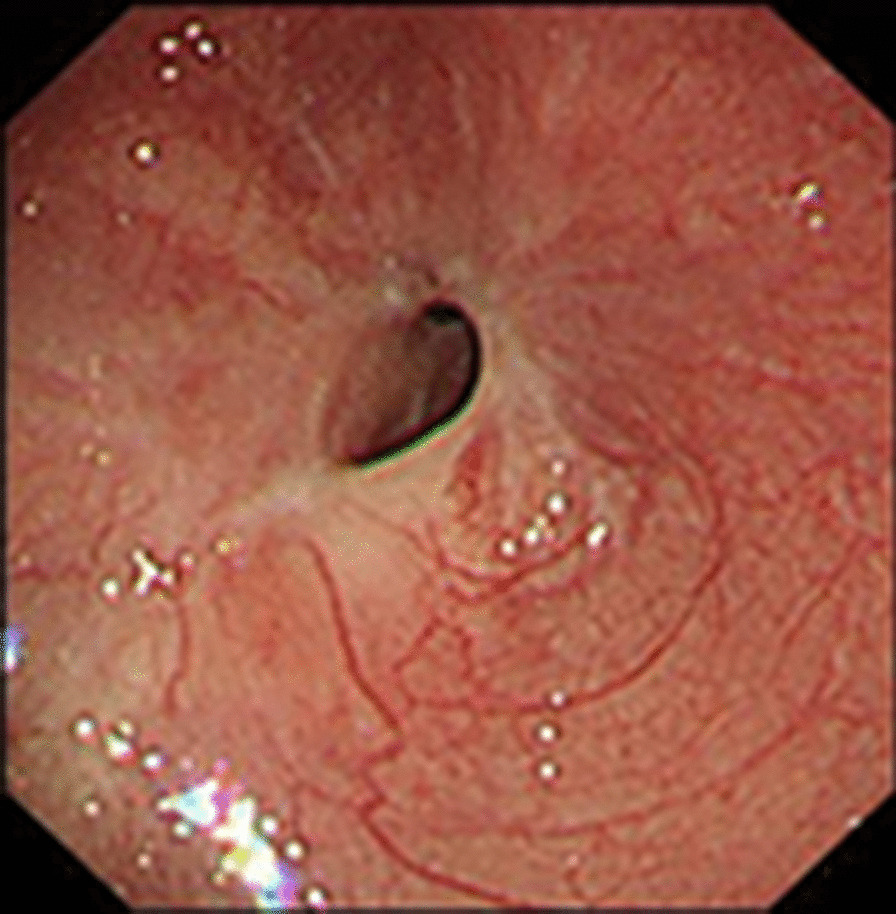
A tight stricture with intact mucosa in esophagus was found during endoscopy

**Fig. 2:**
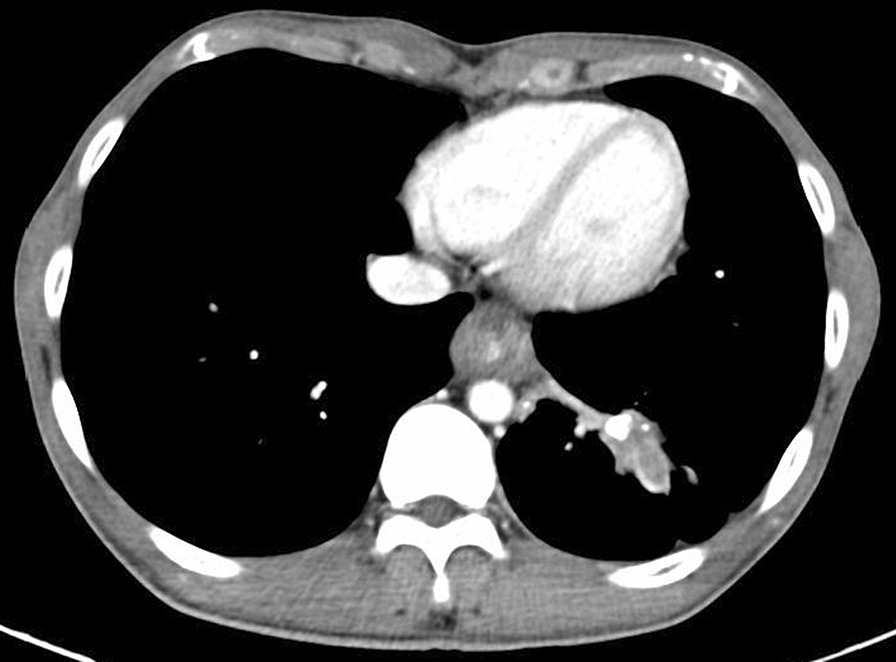
The contrast enhanced CT scan (year of 2018) demonstrated a slight increase in size of the dumbbell-shaped lesion (cross-section)

**Fig. 3:**
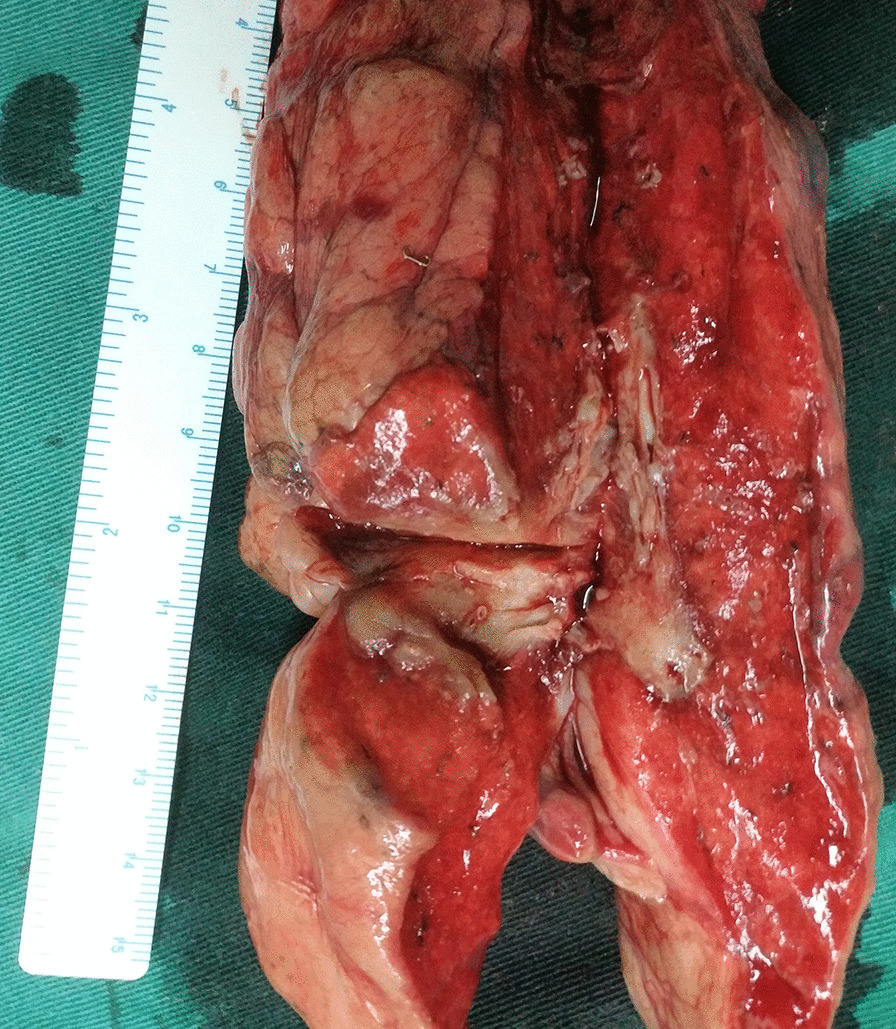
On the cut section of the left lower lobe, a rubbery to firm tumor with a white fibrous was observed

**Fig. 4:**
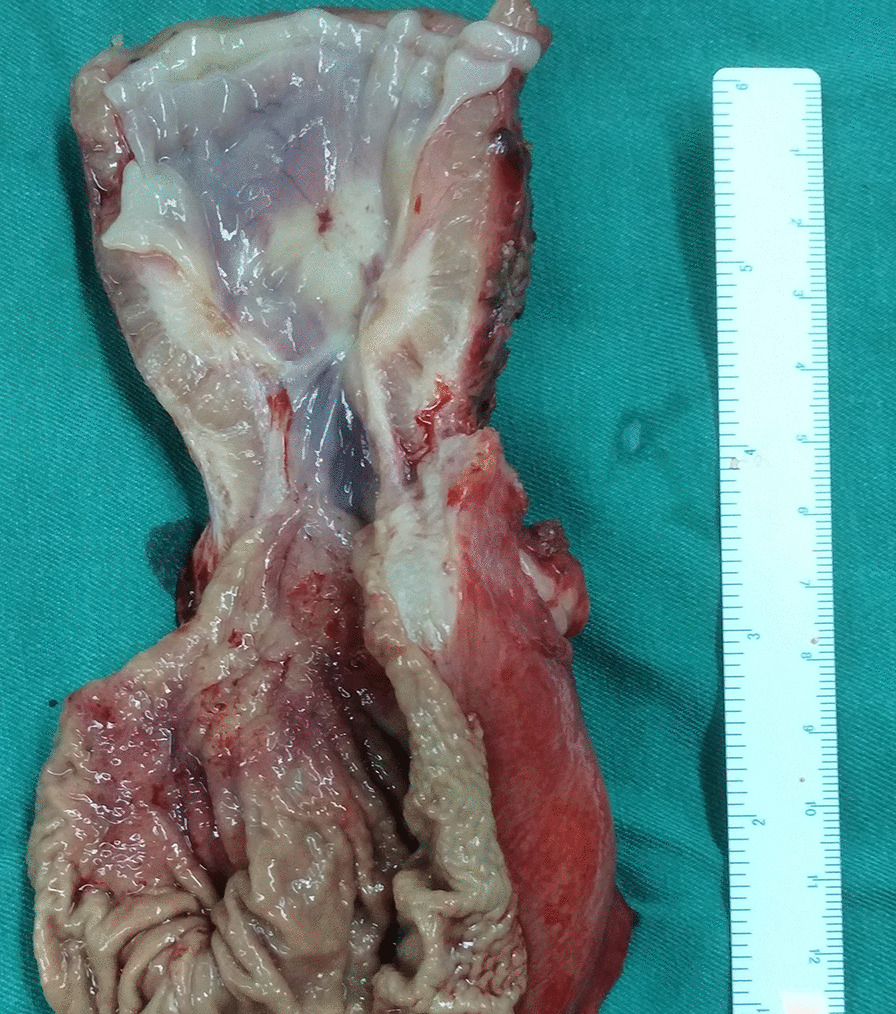
On the cut section of the distal esophagus, a rubbery to firm tumor with a white fibrous was found

**Fig. 5:**
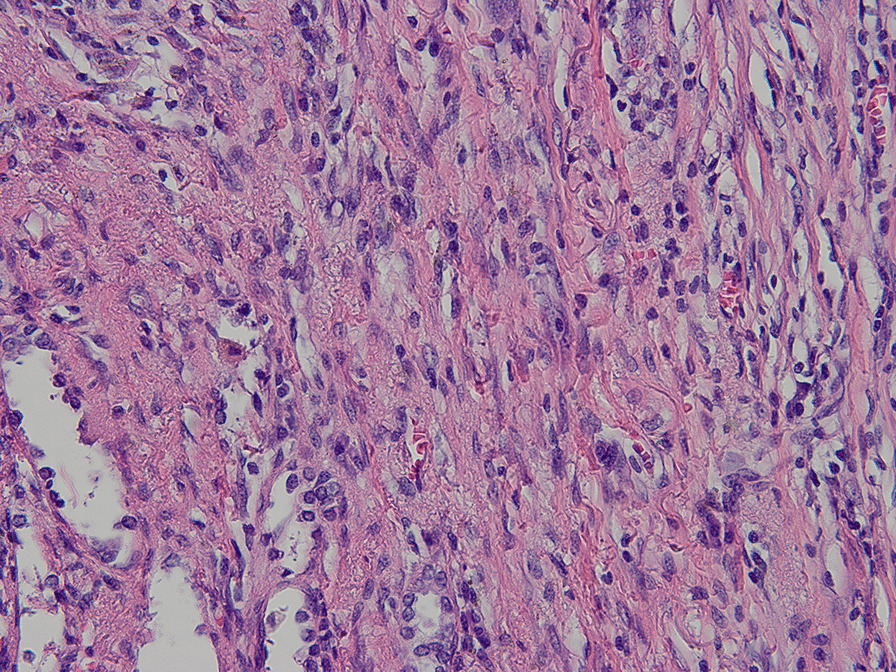
Microscopic examination showed that the tumor contained an inflammatory infiltrate of lymphocytes and multiple proliferating spindle cells

A 29-year-old man presented to our center with progressively dysphagia and weight loss. He denied any history of malignancy and tuberculosis. A tight stricture with intact mucosa in esophagus was found during endoscopy (Fig. 1) and the endoscopy could not be passed across the stricture. Multiple biopsies revealed inflammatory change without malignancy. The CT revealed a dumbbell-shaped lesion between lung and esophagus (Fig. 2). A PET/CT scan revealed an increased FDG uptake of the dumbbell-shaped lesion with mean SUV_max_ of 4.6. After the multiple disciplines discussion, tuberculosis, myofibroblastoma and solitary fibrous tumor were suspected. Given the persistence of dysphagia and definitive diagnosis, the patient elected for surgical resection and underwent partial esophagectomy with gastric tube reconstruction and left lower lobectomy. The tumor epicenter was localized at the lung (Fig. 3) and extensively penetrated downward into the wall of esophagus (Fig. 4). Histologically, the tumor contained an inflammatory infiltrate of lymphocytes (Fig. 5). Immunostaining of the tumor was partially positive for P16, WT-1, and smooth muscle actin (SMA) and negative for anaplastic lymphoma kinase (ALK)-1, CD34, CD117, desmin, and IgG4-positive plasma cells. Eventually, IMT was diagnosed. The postoperative days were uneventful and no recurrence was observed during the follow-up.

## Discussion and conclusions

IMT, firstly reported in 1939 [6], is defined as lesions composed of myofibroblastic spindle cells accompanied by an inflammatory infiltrate of lymphocytes, plasma cells, and eosinophils. It usually happens in the lung, with morbidity from 0.04 to 1.2% [1–3], but also it can be found in omentum, retroperitoneum, pelvis, abdominal soft tissue, head and neck, gastrointestinal track, liver, spleen and larynx. A wide range has IMT been reported, it is mostly found in soft tissue of children and young adults [4]. The etiology of IMT is still controversial, the arguments of which can be divided into two parts: one regarded IMT as neoplasm rather than reactive subset [7], especially the ALK positive IMT was reported to be associated with local recurrence [8], however, rare has IMT been found to metastasis [3]. The other held the causes of IMT include Epstein–Barr virus, human herpes virus eight, reflux, trauma, and overexpression of interleukin 6 [9], which were identified as the unregulated inflammation.

The IMT in this case may have originated in the lung and subsequently invaded the esophagus. To our knowledge, only one case reported the similar case as ours [5]. In Simon et al [5] case, they described a young man (27-year-old) presented with progressive dysphagia for 6 months and diagnosed as left pulmonary IMT invading the gastroesophageal junction. However, their case showed a large mass involving the gastroesophageal junction and left lower lobe without gap between them, meanwhile, the tumor epicenter was localized at the periphery of the resected lung. In our case, dumbbell-shaped lesion between lung and esophagus on CT showed the infiltrative characteristics and neoplasm more clearly. Meanwhile, not only the morphology of this neoplasm was highly consistent with IMT histologically in our case, but also the neoplasm was partially positive for P16, WT-1, and smooth muscle actin (SMA), which are reported to express in IMT and their expression is considered to be an evidence of differential diagnosis among IMT, myofibroblastoma and solitary fibrous tumor [4, 7]. In Simon et al [5] case, however, these stains were negative.

The diagnosis of IMT is difficult preoperatively, and most tumors are diagnosed following the resection. Generally, the manifestations vary from the location of IMT. Fever, dry cough and chest pain were found happened in pulmonary IMT patients [2, 3], and dysphagia, reflux were the appearances of esophageal IMT [10]. Meanwhile, hematemesis and melaena were also reported in the cases of esophageal IMT [11, 12]. However, not all patients will appear the above symptoms and the patients in this study only showed the progressive dysphagia. At the same time, the difference also lies in imaging findings. Pulmonary IMT usually presents with a solitary, peripheral, circumscribed, lobulated mass on chest radiotherapy, however, amorphous with heterogeneous enhancement and punctate calcifications on CT scan. In this case, the dumbbell-shaped lesion initially remind us of the primary complex of tuberculosis, however, the patient didn't have the history and symptom of tuberculosis, nor was the result of terbuculin test negative, therefore, the primary complex of tuberculosis was ruled out. Meanwhile, since IMT has a high cellularity and predominant inflammatory cells, an elevated SUV_max_ will be a pitfall in the differential diagnosis of IMT from malignancy [13, 14]. Endoscopy and radiological examinations are suit for esophageal IMT, but only 77% of the cases were found to be related to histopathology [12, 15, 16]. Variable spindle cell proliferation in a myxoid-to-collagenous stroma, inflammatory infiltrate composed primarily of plasma cells and lymphocytes, variable expression and lack of specificity of myofibroblastic markers can be the evidence of diagnosis of IMT histopathologically [4, 7].

Radical resection is recommended for IMT [2–5, 10, 11]. In this case, the patient received the partial esophagectomy with gastric tube reconstruction and left lower lobectomy and no tumor involvement in margins of proximal of esophagus, stomach and bronchus were found. No recurrence was observed during the follow-up as well. Meanwhile, radical resection and the routine follow-up guarantee the unnecessary of adjuvant therapy administration [2, 3, 5, 10].

In conclusion, we presented a rare case of pulmonary IMT invading the distal of esophagus. IMT is a rare tumor with intermediate malignant potential. Untypical symptom and imaging findings make it difficult to differentiate from other neoplasm. Histopathological findings are of great benefit in the diagnosis of IMT. Completed resection accompanied with closed follow-up is the core of successful treatment.

## Data Availability

All data generated or analyzed during this study are included in this published article.

## Abbreviations

IMT: Inflammatory myofibroblastic tumor
CT: Computed tomography
SMA: Smooth muscle actin
ALK: Anaplastic lymphoma kinase
